# The Effect of Erythropoietin and Its Derivatives on Ischemic Stroke Therapy: A Comprehensive Review

**DOI:** 10.3389/fphar.2022.743926

**Published:** 2022-02-17

**Authors:** Yuanyuan Ma, Zhiyuan Zhou, Guo-Yuan Yang, Jing Ding, Xin Wang

**Affiliations:** ^1^ Department of Neurology, Zhongshan Hospital, Fudan University, Shanghai, China; ^2^ Med-X Research Institute and School of Biomedical Engineering, Shanghai Jiao Tong University, Shanghai, China; ^3^ Department of The State Key Laboratory of Medical Neurobiology, The Institutes of Brain Science and the Collaborative Innovation Center for Brain Science, Fudan University, Shanghai, China

**Keywords:** brain, derivatives, erythropoietin, ischemia, therapy

## Abstract

Numerous studies explored the therapeutic effects of erythropoietin (EPO) on neurodegenerative diseases. Few studies provided comprehensive and latest knowledge of EPO treatment for ischemic stroke. In the present review, we introduced the structure, expression, function of EPO, and its receptors in the central nervous system. Furthermore, we comprehensively discussed EPO treatment in pre-clinical studies, clinical trials, and its therapeutic mechanisms including suppressing inflammation. Finally, advanced studies of the therapy of EPO derivatives in ischemic stroke were also discussed. We wish to provide valuable information on EPO and EPO derivatives’ treatment for ischemic stroke for basic researchers and clinicians to accelerate the process of their clinical applications.

## 1 Introduction

Erythropoietin (EPO) is a glycoprotein hormone mainly produced by the fetal liver and adult kidney and released to the circulation, primarily regulating erythropoiesis in response to hypoxia (Jelkmann, 2011; Rey et al., 2019; Peng et al., 2020). EPO was first described in 1950s and was isolated in 1970s from the urine of patients suffering from aplastic anemia (Inoue et al., 1995; Simon et al., 2019). In 1984, EPO was successfully cloned and expressed in mammalian cells (Inoue et al., 1995; Simon et al., 2019). In 1986, recombinant human EPO (*rh*EPO) was used to treat patients with end-stage renal failure and anemia, elevating the hemoglobin concentration of the plasma in 9 out of 12 patients (Winearls et al., 1986). Then, biologically active *rh*EPO was produced in Chinese hamster ovary cells, which were generally used for the large-scale manufacture of EPO analogous erythropoiesis-stimulating agents (ESAs) (Recny et al., 1987; Jelkmann, 2013). In 1989, *rh*EPO was approved by the Food and Drug Administration of the United States of America for clinical treatment of anemia associated with chronic renal failure due to insufficient EPO production and showed improvement in the life quality of patients (Wright et al., 2015; Suresh et al., 2019). Since its clinical availability in 1990s, apart from chronic kidney diseases, *rh*EPO has been widely used to treat all sorts of anemias induced by different etiologies such as infection and chemotherapy for various cancers (Hernández et al., 2017; Simon et al., 2019). Numerous *in vitro* and *in vivo* studies demonstrated that *rh*EPO was a potential therapeutic approach to treat a variety of diseases, especially neurological diseases including Alzheimer’s disease (AD), Parkinson’s disease (PD), amyotrophic lateral sclerosis, spinal cord injury, epilepsy, hypoxia, traumatic brain injury, subarachnoid hemorrhage, and ischemic stroke (Hernández et al., 2017; Wei et al., 2017; Blixt et al., 2018; Merelli et al., 2019; Rey et al., 2019; Simon et al., 2019). Currently, EPO is one of the most popular biopharmaceutical products worldwide (Hernández et al., 2017).

## 2 Structure, Expression, and Function of EPO and EPO Receptors in the Central Nervous System

### 2.1 Structure of EPO

**FIGURE 1:**
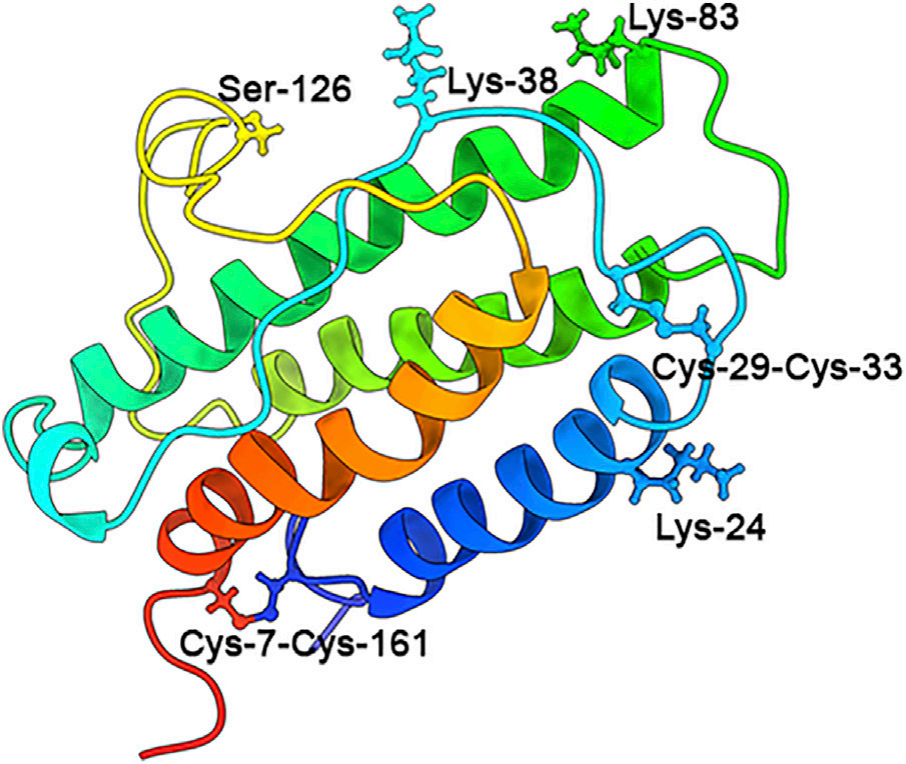
Human EPO structure. The globular three-dimensional structure: four amphipathic α helices, two β-sheets, and two intra-chain disulfide bridges (Cys-7-Cys-161 and Cys-29-Cys-33) (Bunn, 2013; Jelkmann, 2013; Sinclair, 2013; Ostrowski and Heinrich, 2018). The three N-linked polysaccharide groups are Lys-24, Lys-38, and Lys-83, and the one O-linked group is Ser-126 (PBD ID: EER).

The human EPO belongs to the superfamily of type I cytokines and is present in all vertebrates (Brines and Cerami, 2005; Kunze and Marti, 2019). Human EPO is composed of 166 amino acids presenting with a globular three-dimensional structure and forms four amphipathic α-helices, two β-sheets, and two intra-chain disulfide bridges (Cys-7-Cys-161 and Cys-29-Cys-33) (Jelkmann, 2013; Ostrowski and Heinrich, 2018). Similar to *rh*EPO, human EPO has about 40% carbohydrate (w/w), consisting of three N-linked polysaccharide groups and one O-linked group (Bunn, 2013). In *rhEPO*, the three N-linked polysaccharide groups are at positions Asn-24, Asn-38, and Asn-83, and one O-linked group is at residue Ser-126 (Elliott et al., 2003; Bunn, 2013; Sinclair, 2013). However, in human EPO, the amino acid residues are Lys-24, Lys-38, and Lys-83 (PBD ID: EER) (Figure 1). Notably, the glycosylation distribution makes a great heterogeneity to the maturity of EPO and regulates EPO pharmacokinetic and pharmacodynamic properties. In addition, the glycosylation distribution controls the interaction of EPO with the receptors and modulates its biological activity (Sinclair, 2013; Castillo et al., 2018). The weight of human EPO is 30.4 KDa, while that of recombinant EPO is approximately 34 KDa (Asadi et al., 2013; Jelkmann, 2013; Rama et al., 2019). Figure 1.

### 2.2 EPO Production

In humans, the main EPO-producing organ changes throughout the life (Rey et al., 2019). During fetal development, EPO is produced in the liver, which is the organ producing red blood cells. After birth and during adulthood, renal tubular interstitial cells of the kidney gradually become the major region of EPO production and secretion (Jelkmann, 2001). Beyond fetal liver and adult kidney, which accounted for the majority of circulating EPO in humans, EPO could be locally produced and released by cells of various organs and tissues including the heart, spleen, bone marrow, lungs, testis, ovaries, retina, and CNS (Ostrowski and Heinrich, 2018; Suresh et al., 2019).

These non-erythroid tissues produced about 15%–20% of the total EPO (Bunn, 2013). It was noted that EPO mRNA could be detected in rodents, monkeys, and human brains (Digicaylioglu et al., 1995; Marti et al., 1996). In the human brain, astrocytes, oligodendrocytes, neurons, and endothelial cells from the cortex, hippocampus, amygdala, and midbrain were capable to produce EPO in a paracrine or autocrine manner (Marti, 2004; Ogunshola and Bogdanova, 2013; Simon et al., 2019). In addition, EPO could be detected in the cerebrospinal fluid (CSF) of neonates and adults (Marti, 2004). Hypoxia could induce EPO production. The EPO expression was increased by binding of the hypoxia-inducible factor (HIF) to the hypoxic responsive element located downstream of the coding region under hypoxic conditions (Semenza, 2009; Kobayashi et al., 2017; Anusornvongchai et al., 2018). The HIF is a heterodimer between HIF-α (HIF-1α, HIF-2α, or HIF-3α) and HIF-1β (or ARNT). When the oxygen level was reduced, HIF-α was stabilized, and HIF-2α was increased in renal EPO-producing cells, upregulating the EPO gene expression (Semenza, 2009).

Previous studies showed that the number of cells producing EPO in the kidney was increased and resulted in the enhanced EPO upon hypoxia (Suresh et al., 2019). When the erythrocyte level was reduced, the renal tubular interstitial cells sensed relative hypoxia and synthesized and released EPO into the plasma in a classic endocrine manner. EPO then migrated to the bone marrow and promoted erythropoiesis to enhance the oxygen binding and transport capacity, which has been the principal function of EPO since its discovery (Jelkmann, 2011; Peng et al., 2020). High serum EPO levels have been demonstrated to be linked to fractures in elderly male population (Rauner et al., 2021). However, previous studies found that the EPO expression was decreased in the hippocampus of aging rats compared to that of the young rats (Li et al., 2016). Oxidative stress could be the main reason for the decline of brain EPO in the aging rats, and the decrease of HIF-2α stability was involved in the decline (Li et al., 2016). Recently, a clinical study reported that plasma EPO was increased in both young and old people after normobaric hypoxia of 180 min. The amount of EPO was higher in young people during the same normobaric hypoxia than that in old people (Törpel et al., 2019). What the exact roles of the changes of the EPO level during pathophysiological conditions including aging remain further explored.

In addition to hypoxia, EPO production could be induced in response to a number of other challenges or instants including anemia, high altitude, mechanical damage, infection, metabolic stress, elevated temperature, intense neural activity, enriched environment, and ischemic stress (Pugh and Ratcliffe, 2017; Ostrowski and Heinrich, 2018). In humans, the circulation half-life of kidney-derived EPO is 5–6 h due to high levels of glycosylation (Peng et al., 2020). The normal range of EPO in the plasma of healthy individuals is 10–20 (mIU/ml) (Bunn, 2013). In patients with middle cerebral artery occlusion (MCAO), serum EPO levels peaked 2.6-fold at day 7 after MCAO and remained elevated until day 30 post stroke (Ehrenreich et al., 2002). The serum EPO at the acute stage was positively correlated with the severity of stroke, but an increase in EPO levels between the acute stage and 3 months after ischemic stroke was associated with better functional outcomes evaluated by the Scandinavian Stroke Scale (Åberg et al., 2016).

### 2.3 The Structure and Expression of EPO Receptors

**FIGURE 2:**
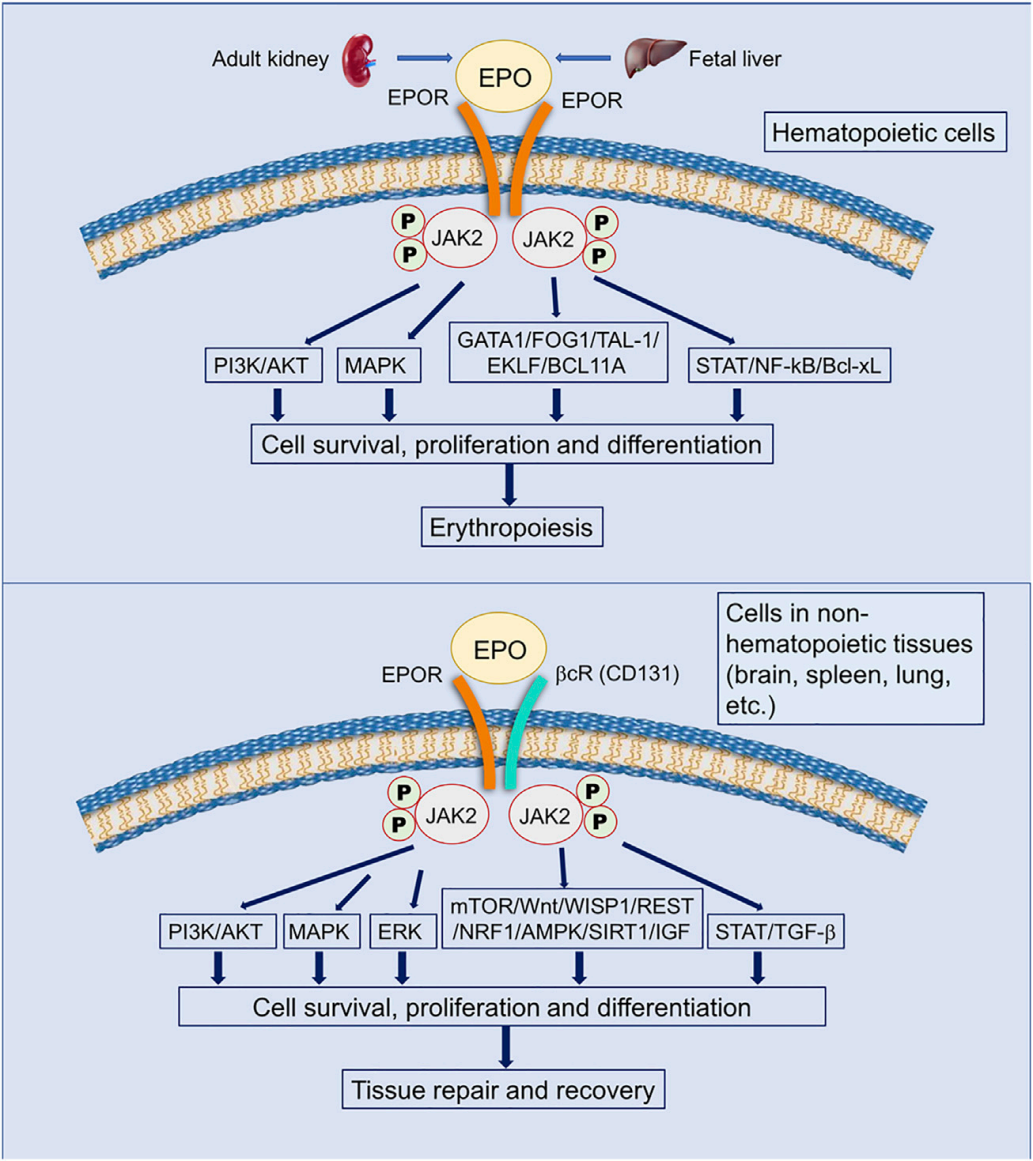
EPO and EPO receptors. EPO has been demonstrated to interact with two classic receptors to initiate its pleiotrophic effects. A homodimeric receptor with two units of EPOR (yellow structures) is expressed on hematopoietic cells, while a heterodimeric receptor with one EPOR and one βcR (CD131) unit (yellow and cyan structure) is expressed on cells in non-hematopoietic tissues such as the brain, spleen, and lungs. EPO activates different intracellular pathways through EPORs, leading to anti-apoptotic gene expression and the inhibition of pro-apoptotic genes. These actions allow for cell survival, proliferation, and differentiation. Activation of the homodimeric EPOR/EPOR leads to erythropoiesis, while activation of the heterodimeric EPOR/βcR (CD131) leads to tissue repair and recovery (Socolovsky et al., 2001; Tsiftsoglou, 2021; Maiese, 2015; Gyetvai et al., 2017; Othman et al., 2018; Jarero-Basulto et al., 2020; Peng et al., 2020; Jacobs et al., 2021; Larpthaveesarp et al., 2021). The JAK/STAT (Janus kinase/signal transducer and activator of transcription) pathway is involved in many vital cellular processes, including cell growth, differentiation, proliferation, and regulatory immune functions; PI3K/AKT (Phosphatidylinositol-4,5-bisphosphate 3-kinase/protein kinase B) is activated by numerous genes and improves cell proliferation during erythropoiesis in hypoxia; ERK/MAPK (extracellular signal-regulated kinase/Mitogen-activated protein kinase) is the key signaling pathway that regulates a wide variety of cellular processes, including proliferation, differentiation, apoptosis, and stress responses; GATA1, FOG1, TAL-1, and EKLF (Erythroid Kruppel-like Factor, also called KLF1) are master transcriptional regulators of erythropoiesis; BC11A, transcriptional repressor B-cell lymphoma/leukemia 11A, is a transcriptional repressor of erythropoiesis; TGF-β, transforming growth factor-β, regulates cell growth and differentiation, apoptosis, etc.; NF-kB, the nuclear factor kB, could regulate the expression of genes involved in cell proliferation, migration, and apoptosis; Bcl-xL, B-cell lymphoma extra-large, is an anti-apoptotic Bcl-2 protein; mTOR, mammalian target of rapamycin, is a protein kinase regulating cell growth, survival, metabolism, and immunity; Wnt, wingless-type, the ligand of Wnt-signaling, are unique directional growth factors that contribute to both cell proliferation and polarity; WISP1, Wnt1-inducible signaling pathway protein 1, a target of Wnt1, mediates cell proliferation and apoptosis, etc.; REST, the repressor element 1-silencing transcription, is a repressor of neuronal genes during embryonic development and regulates a network of genes that mediate cell death in the aging human brain (Lu et al., 2014); NRF1, nuclear factor erythroid 2-related factor-1, is a endoplasmic reticulum-bound transcription factor that regulates protein homeostasis; AMPK, AMP-activated protein kinase, plays a major role in regulating cellular energy balance; SIRT1, silent information regulator 2 homolog 1, is a protein deacetylase that mediates cell self-renewal; IGF, insulin-like growth factor, induces the signaling networks, which are vital in modulating multiple fundamental cellular processes, such as cell growth, survival, proliferation, and differentiation.

It has been widely known that EPO exerts its functions *via* binding to EPO receptors (EPORs) (Suresh et al., 2019), which activate key signal pathways controlling cell survival, proliferation, differentiation, apoptosis, death, and neuroprotection (Hernández et al., 2017; Suresh et al., 2019; Urena-Guerrero et al., 2020) (Figure 2). EPORs belong to the class I cytokine receptor superfamily and consist of a WSXWS motif in the extracellular domain of 225 amino acids, a single transmembrane domain of 23 amino acids, and a cytoplasmic domain of 235 amino acids. The cytoplasmic domain, lacking tyrosine kinase activity and associated with Janus kinase (JAK), forms complexes that determine EPORs are homodimeric, heterodimeric, or heterotrimeric (Liongue et al., 2016; Suresh et al., 2019; Urena-Guerrero et al., 2020). To date, four isoforms of EPORs including the EPOR/EPOR (EPOR), EPOR/β-common receptor (βcR), EphrinB4 receptor (EphB4), and cytokine receptor-like factor 3 (CRLF3) were discovered in animals (Ostrowski and Heinrich, 2018; Suresh et al., 2019; Urena-Guerrero et al., 2020) Figure 2.

#### 2.3.1 EPOR Expression

EPOR is the classical EPO receptor, which is a homodimeric molecule weighing 66 KDa. EPOR is expressed at the highest level on erythroid progenitor cells and promotes cell proliferation, differentiation, and survival in mature red blood cells (Suresh et al., 2019). EPO could regulate the expression of its own receptor *via* binding to its receptor on erythroid progenitor cells (Suresh et al., 2019). The interaction between EPO and EPOR resulted in the increased erythroid transcription factors including GATA1 and the basic-helix-loop-helix protein, TAL1, which in turn transactivated the EPOR expression (Suresh et al., 2019). Apart from erythroid progenitor cells, EPOR was also expressed in the CNS and played a crucial role for the normal development of the brain (Ostrowski and Heinrich, 2018; Schneider Gasser et al., 2019). In the development of mouse brain, EPOR was expressed in the neural tube (in radial glial cells) as early as embryonic 8 (E8) at levels comparable to the adult hematopoietic tissue (Knabe et al., 2004). The CA1 region of the hippocampus was found to be among the highest expression of EPOR in the brain (Wakhloo et al., 2020). Both EPO and EPOR expressions could be further elevated by the complex cognitive challenge paradigm (Wakhloo et al., 2020). In addition, EPOR was found to be abundantly present in specific brainstem nuclei and played an important role in the central control of ventilation across development and adulthood in rodents (Schneider Gasser et al., 2019). In the developing human embryo, EPOR was detected as early as 7–8 weeks in neurons and astrocytes of the brain and spinal cord (Juul et al., 1998; Juul et al., 1999). In EPOR-knockout mice, the overall number of neuronal progenitor cells was reduced as well as neurogenesis (Tsai et al., 2006). Under normal/healthy conditions, the EPOR expression in adult nervous systems remained very low (Hernández et al., 2017). A variety of factors such as environmental enrichment, ambient heat, or mild episodes of hypoxia could increase the EPOR expression and furthermore protected neurons toward following injuries including severe ischemia (Sanchez et al., 2009; Larpthaveesarp et al., 2016; Hernández et al., 2017; Ostrowski and Heinrich, 2018). There existed the age-associated expression of EPO and its receptor EPOR in rat spiral ganglion neurons and its association with neuronal apoptosis and hearing alterations (Zhong and Zhang, 2017). Compared to the infant, the adult and aged rats showed increased EPOR expressions in spiral ganglion neurons in the inner ears (Zhong and Zhang, 2017), indicating that the age-associated increased expression of EPOR exerted a role in neuroprotection when necessary as in presbycusis. Furthermore, induction of the EPOR expression in non-hematopoietic tissues following injuries was shown to be correlated with tissue protective effects of EPO administration in animal models of a variety of diseases including ischemic stroke (Castañeda Arellano et al., 2014; Larpthaveesarp et al., 2016).

#### 2.3.2 EPOR/βcR

EPOR/βcR is the non-canonical receptor expressed in non-hematopoietic tissues including the heart, retina, pancreas, kidney, and brain. EPOR/βcR mediates EPO-induced, erythropoiesis-independent, tissue-protective effects (Cantarelli et al., 2019; Guglielmo et al., 2019). EPOR/βcR is a heterodimer, of which one subunit is the canonical EPOR receptor. Another subunit is βcR, also known as CD131, that is shared by type 1 cytokines including GM-CSF, IL-3, and IL-5 (Murphy and Young, 2006; Hernández et al., 2017). Unlike EPOR, EPOR/βcR had much lower affinity for EPO. Therefore, it required a higher EPO concentration than that in the circulating serum to initiate tissue-protective effects (Cantarelli et al., 2019). Non-erythroid cells expressing EPOR/βcR included endothelial cells, tumor cells, monocytes, macrophages, dendritic cells, mast cells and lymphocytes, skeletal muscle myoblasts, and neural cells (Lisowska et al., 2010; Kimáková et al., 2017; Annese et al., 2019; Suresh et al., 2019; Torregrossa et al., 2019). In the CNS, many types of cells including neurons, astrocytes, microglia, and endothelial cells expressed EPOR/βcR (Ostrowski and Heinrich, 2018). Using βcR-deficient mice, a study demonstrated that EPO mediated neuroprotection through EPOR/βcR after spinal cord injury (Ostrowski and Heinrich, 2018). The EPO derivatives including neuro-EPO, carbamylated erythropoietin (CEPO), and asialo-erythropoietin (asialo-EPO) preferentially bound to EPOR/βcR instead of the classical homodimer EPOR (Jerndal et al., 2010; Castillo et al., 2018). However, evidence of the direct interaction between the subunits EPOR receptor and βcR is still lacking (Suresh et al., 2019). A study using biophysical analyses on the silico docking studies showed that the extracellular domains of EPOR and βcR did not directly interact with each other in the presence or absence of EPO (Cheung Tung Shing et al., 2018). Therefore, the role of βcR in response to EPO remains uncertain and needs to be further studied.

#### 2.3.3 EphB4

**FIGURE 3:**
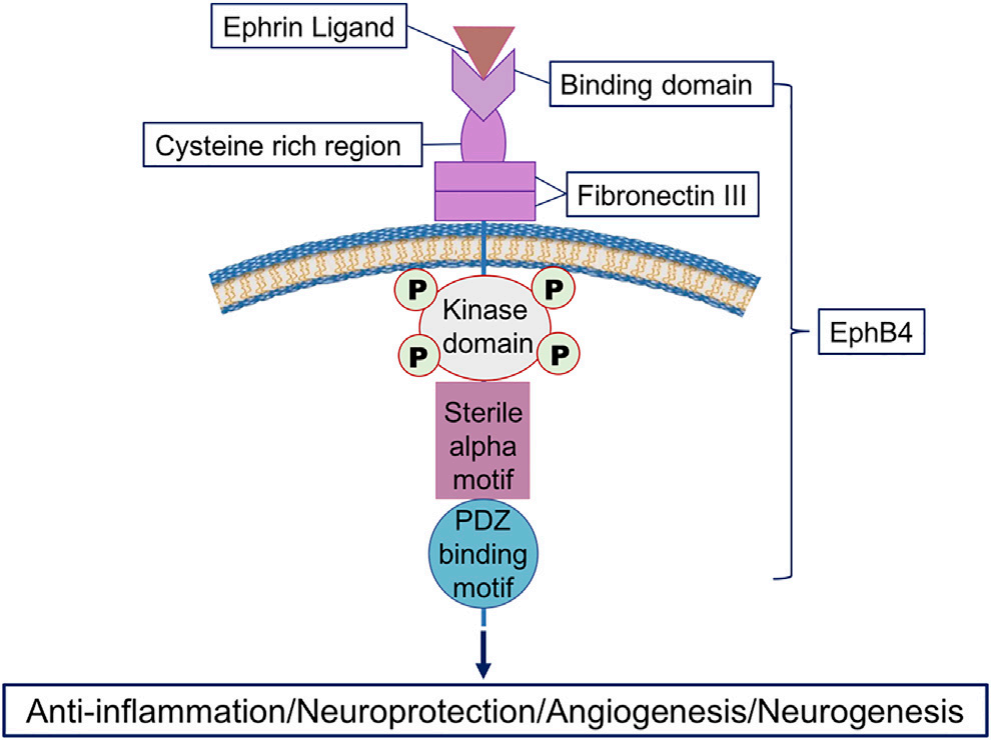
Illustration of the interaction between the ephrin ligand and the receptor EphB4. EphB4 has an extracellular region and an intracellular region. The extracellular region includes an ephrin-binding domain, a cysteine-rich region, and two fibronectin type III repeats, while the intracellular region contains a tyrosine kinase domain, a sterile alpha motif domain, and a PDZ-binding motif. The ephrin ligand, for example, ephrin4 binds to EphB4 to initiate multiple effects including anti-inflammation, neuroprotection, angiogenesis, and neurogenesis during pathophysiological conditions (Salgia et al., 2018; Chen Y. et al., 2019; Elgebaly, 2020).

EphB4 is a member of the largest subfamily of receptor tyrosine kinases that typically mediate contact dependent cell-to-cell communication through interacting with membrane-bound ephrin ligands (Kania and Klein, 2016) (Figure 3). EphB4 is different from other ephrin receptors (Eph) due to the containment of an isoleucine instead of a tyrosine at position 48 in the hydrophobic cavity (Ostrowski and Heinrich, 2018). EphB4 was an EPO receptor that triggered downstream signaling through STAT3 and promoted EPO-induced tumor growth and progression (Pradeep et al., 2015). Additionally, rat cortex neurons co-expressed EPOR and EphB4, which were activated by EPO (Ostrowski and Heinrich, 2018). EphB4 was expressed in the mammalian nervous system and was involved in regulating adult neurogenesis and gliogenesis in the subgranular zone (SGZ) of the hippocampus. After ischemic stroke, the interaction between ephrin ligands, for example, ephrin2 and EphB4 could reduce brain edema and infarct size, attenuate inflammation, and improve motor function (Elgebaly, 2020). However, whether EPO interacts with EphB4 to initiate protective effects in ischemic stroke is still unclear (Ashton et al., 2012; Liu et al., 2017) Figure 3.

#### 2.3.4 CRLF3

The human CRLF3 gene is located on chromosome 17. The protein with 442 amino acids of CRLF3 belongs to class I helical cytokine receptors. CRLF3 mediated pleiotropic cellular reaction to injuries and diverse physiological challenges (Ostrowski and Heinrich, 2018; Hahn et al., 2019). CRLF3 was expressed in various tissues and functioned as a cell protective receptor for EPO. Additionally, CRLF3 was essential for *rh*EPO-mediated neuroprotection in locust brain neurons under hypoxia (Hahn et al., 2017; Hahn et al., 2019). However, the role of CRLF3 as a neuroprotective receptor in mammals and humans (Ostrowski and Heinrich, 2018) or a protective function in ischemic stroke needs to be explored.

In general, EPOR and EPOR/βcR mediate EPO’s protective and regenerative functions in the CNS (Urena-Guerrero et al., 2020). The downstream signaling was first initiated by JAK2 phosphorylation, followed by STAT phosphorylation and activation, and others including PI3K/AKT and ERK1/2 pathways. It was noted that phosphorylated STAT5 subunits STAT5A and STAT5B dimerized and translocated into the nucleus to activate the selected gene expression. These genes such as Bcl-xL, NF-kB, and TGF-β were related to cell proliferation, apoptosis, and differentiation (Socolovsky et al., 2001; Ostrowski and Heinrich, 2018; Suresh et al., 2019; Jarero-Basulto et al., 2020; Tsiftsoglou, 2021). Different isoforms of EPORs were expressed in different neural cell types (Rey et al., 2019). However, whether homodimeric EPOR or the heteromeric complex EPOR/βcR relay the EPO signaling in the responsive cells is not addressed (Ostrowski and Heinrich, 2018; Rey et al., 2019).

Future studies with specific EPO-mimetic ligands are needed to elucidate the roles of EPORs in EPO-mediated neuroprotection under ischemia. Developing new isoform-selective drugs may promote a more specific therapy targeting EPORs for ischemic stroke.

### 2.4 The Function of Endogenous EPO

In addition to its key physiological function of regulating erythropoiesis, emerging evidence has shown that EPO possesses multiple non-hematopoietic biological functions such as anti-apoptosis, antioxidant, neuroprotection, neurogenesis, angiogenesis, and immunomodulation (Nekoui and Blaise, 2017; Cantarelli et al., 2019; Guglielmo et al., 2019; Rama et al., 2019; Rey et al., 2019).

#### 2.4.1 The Function of Endogenous EPO in the Normal CNS

Biologically active EPO synthesized in the mouse brain played crucial roles in neurodevelopment and in neurotransmission modulation (Masuda et al., 1994; Digicaylioglu et al., 1995). EPOR was found expressed in the developing and adult mammal brain and displayed a higher level in neural progenitor cells than that in mature neurons (Chen et al., 2007; Noguchi et al., 2007; Sollinger et al., 2017). A study using EPOR-null mice showed that endogenous EPO was required for normal neural progenitor cell proliferation independent of injury or ischemia, contributing directly to brain development, maintenance, and repair (Chen et al., 2007). STAT5, STAT3, REST, and NRF1 were the downstream factors of EPO signaling mediating epigenetic and transcription networks, which were associated with differentiation and plasticity in fetal neural progenitor cells (Sollinger et al., 2017). Using *in situ* hybridization assay and immunofluorescence techniques, EPOR was detected in specific brainstem nuclei associated with central sensitivity of CO2 and control of ventilation in the ventrolateral medulla. This indicated that EPO signaling played an important role in the central control of ventilation across development and adulthood in rodents (Schneider Gasser et al., 2019). In young healthy mice, 3-week EPO administration was associated with the increased numbers of pyramidal neurons and oligodendrocytes in the hippocampus (Hassouna et al., 2016). A microarray analysis demonstrated that EPO increased the re-myelination tendency of oligodendrocytes by inducing the insulin-like growth factor (IGF) expression and increasing lipid metabolism (Gyetvai et al., 2017).

#### 2.4.2 The Function of Endogenous EPO During Ischemic Brain Injury

Recent studies have demonstrated that EPO elicited beneficial effects in cerebral ischemia, especially in preventing ischemic neuronal injury (Yoo et al., 2017; Komnig et al., 2018). Both circulating and tissue-derived EPO and the locally produced EPO mediated the neuroprotection function in non-hematopoietic tissues (Ostrowski and Heinrich, 2018). EPO and its receptors were increased in the brain in both rodents and mammals exposed to ischemic or hypoxic damage (Shingo et al., 2001; Suzuki et al., 2001). Endogenous EPO protected hypoxic astrocytes and oligodendrocyte precursor cells *in vitro* and enhanced ischemic neuron survival *in vivo* (Sakanaka et al., 1998; Bernaudin et al., 2000; Kato et al., 2011). In human ischemic or hypoxic brains, EPO and its receptors were upregulated (Sirén et al., 2001). In patients who underwent carotid endarterectomy, brain EPO was enhanced during carotid clamping (Carelli et al., 2016). In rats that underwent hypoxic precondition for 3 or 21 days before permanent MCAO surgery, brain EPO was elevated associated with the reduced infarct volume at 24 h after ischemia (Darlington et al., 2021). A recent study has shown that EPO promoted a synaptic protein Synapsin1 and PSD95 expression, reduced axonal injury, and restored axonal density, contributing to improving electrophysiological properties of synapses and spatial memory performance after hypoxia-ischemia in neonatal rats (Xiong et al., 2019). These results suggested that EPO signaling played a crucial role for endogenous neuroprotection under hypoxia or ischemia (Terraneo and Samaja., 2017). An *in vitro* study showed that EPO attenuated ischemic vascular injury partially through direct modulation of AKT phosphorylation to prevent DNA fragmentation, resulting in reduced mitochondrial membrane depolarization and cytochrome c release (Chong et al., 2002). The mechanism of EPO induced neuroprotective effects that included the bind between EPO and the receptor, followed by activating PI3K, AKT, mTOR, Wnt, WISP1, AMPK, and silent mating-type information regulation 2 homolog 1 (SIRT1) (Maiese, 2015; Othman et al., 2018). However, the mechanism of EPO-induced neuroprotection in ischemic stroke remains vague, and further studies are warranted.

## 3 EPO for the Ischemic Stroke Therapy

### 3.1 EPO Therapy in Pre-Clinical Studies of Ischemic Stroke

EPO could cross the blood–brain barrier (BBB) by receptor-mediated transcytosis (Dame et al., 2001; Fu et al., 2010; Rodríguez Cruz et al., 2010; Yoo et al., 2017). Therefore, exogenous administration of EPO may enhance its constitutive effects in the brain. Numerous studies evaluated the effect of *rh*EPO therapy and showed encouraging results at acute and delayed stages of ischemic stroke. Generally, *rh*EPO was given systemically once or multiple times.

#### 3.1.1 Different Doses, Routes, and Times of *rh*EPO Treatment in Ischemic Stroke Models

The interaction between EPO and its receptor not only regulates erythropoiesis in response to hypoxia but also exerts protective functions in non-hematopoietic tissue, which express EPORs with low affinity for EPO (Cantarelli et al., 2019). Based on different isoforms of EPORs with different structures and functions, EPO concentration for tissue protection is 100–1,000 times higher than that needed for red blood cell production (Teramo et al., 2018). A typical dose of *rh*EPO 100 IU/kg could make serum EPO trigger EPORs for approximately 24 h (Brines and Cerami, 2008). In terms of ischemic stroke, doses of *rh*EPO from 500 to 5000 IU/kg IV given multiple times (starting at 6 h and repeated at 24 and 48 h) reduced the infarct volume and neurological impairment after 28 days of cerebral ischemia in rats (Wang et al., 2007). However, studies also showed that the IV injection of *rh*EP O 10 min before or immediately after MCAO with a dose of 1000 IU/kg once did not reduce the infarct volume after 72 h of focal ischemia in rodents (Zhang et al., 2010; Ratilal et al., 2014). Using a similar MCAO model in mice, *rh*EPO (5,000 IU/kg) was given IV immediately and the following day after reperfusion, the survival rate and neurological function were improved after 7 days of cerebral ischemia (Chen GH. et al., 2019). With the identical dose (5,000 IU/kg) but with the given Intraperitoneal injection (IP) at different time points, *rh*EPO administration also improved neurological outcomes after 14 days of cerebral ischemia. The protective mechanism was related to enhance the pro-survival endoplasmic reticulum to nucleus signaling 1 (IRE1a) activation, compromise the pro-apoptotic protein kinase R (PKR)-like endoplasmic reticulum kinase (PERK) branch of the unfolded protein response, and facilitate oligodendrogenesis (Wang et al., 2017a; Habib et al., 2019a; Habib et al., 2019b). Similarly, given subcutaneously from 0.5 to 48 h after MCAO in rats, *rh*EPO of 5,000 IU/kg reduced the brain infarct area after 72 h of cerebral ischemia through suppressing the innate immune response to inflammation, oxidative stress, microRNAs (miR-223/miR-30a/miR-383), and mitogen-activated protein kinase (MAPK) family signaling (Yuen et al., 2017). When given *via* an intra-artery route, even with a lower dose (800 IU/kg) at the beginning after reperfusion, *rh*EPO alleviated the infarct volume, brain edema, and improved neurobehavioral outcomes at 2 and 24 h after MCAO (Wang et al., 2015). The alleviation of BBB disruption was associated with reduced degradation of claudin-5 and occludin and reduced the microvessel matrix metalloproteinase-2/9 (MMP-2/9) expression and activity after *rh*EPO treatment (Wang et al., 2015). Beyond the acute ischemia, delayed *rh*EPO treatment, which started 1 week after MCAO, reduced the infarct volume and improved behavioral outcomes in neonatal rats after 1 month of ischemia (Larpthaveesarp et al., 2016). EPO concentrations in CSF derived from IV injection were six times higher than those derived from the IP injection (Zhang et al., 2010). Therefore, among these administration approaches, IV with multiple injections seems a better choice, which has a great potential for clinical application Supplementary Table S1.

In addition to the above routes, intranasal delivery was also successfully exploited. Intranasal-delivered *rh*EPO could go through BBB and subsequently move to the brain parenchyma (Merelli et al., 2018; Rey et al., 2019). Compared to the systemic approach, intranasal delivery bypassed the first-pass effect of the liver and achieved potentially therapeutic levels of drugs including EPO in the CNS (Alcalá Barraza et al., 2010). Intranasal delivery of *rh*EPO was 10 times faster than that of the IV route (Teste et al., 2012), achieving therapeutic effects in a mouse and a rat model of cerebral ischemia (Yu et al., 2005; Fletcher et al., 2009; Merelli et al., 2011). A single intranasal delivery of *rh*EPO (1 h of post-injury) with a low dose of 500 or 1000 IU/kg provided histological neurorepair in the CA1 hippocampal region after ischemic brain injury (Macias-Velez et al., 2019). Thus, *rh*EPO intranasal delivery is an alternative and promising approach for ischemic stroke therapy.

#### 3.1.2 The Mechanism of EPO in Ischemic Stroke

**FIGURE 4:**
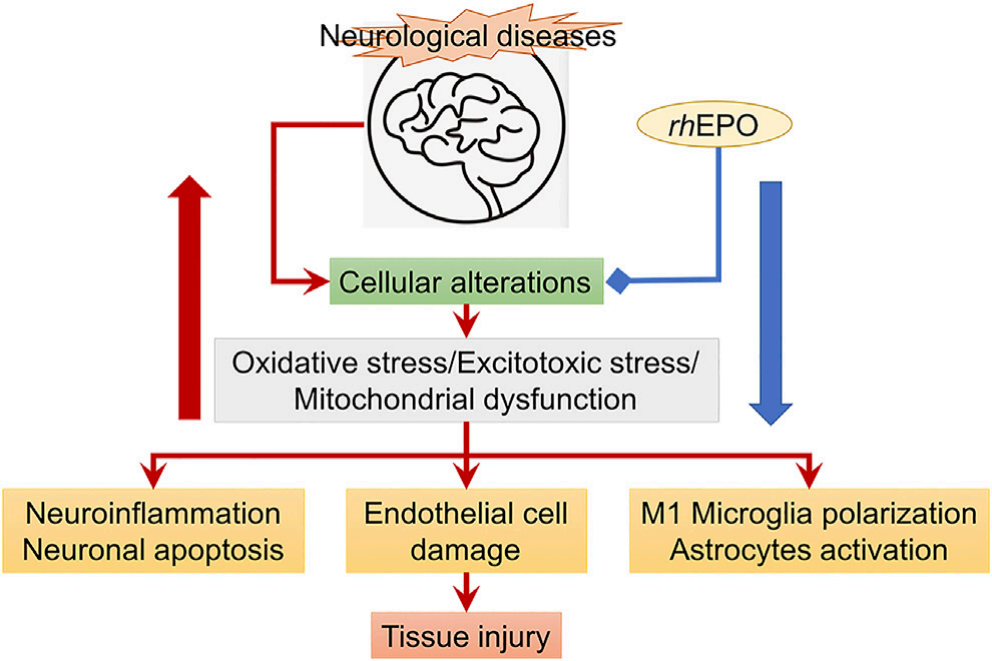
*rh*EPO treatment (blue line) attenuates cellular alterations (read arrows) resulted from ischemic stroke. The red big arrow refers to increased cellular alterations, while the blue big arrow refers to inhibition of cellular alterations and the subsequent tissue injury.

The therapeutic effects of EPO treatment for ischemic stroke have been widely studied (Figure 4). However, the mechanism underlying the therapeutic effects is not fully delineated, particularly at the delayed stage of cerebral ischemia. Several studies have shown that exogenous administration of *rh*EPO could attenuate BBB disruption after cerebral ischemia probably through reducing lipid peroxidation in the brain, downregulating the vascular endothelial growth factor receptor-2 (VEGFR-2) expression along the penumbra region and alleviating the MMP-2 and MMP-9 activity in ischemic microvessels (Li et al., 2007a; Bahcekapili et al., 2007; Chi et al., 2008; Wang et al., 2015). A study using a platelet-rich thrombus-induced stroke model in mice showed that *rh*EPO treatment only reduced neuronal apoptosis and BBB permeability in young mice at 24 h after ischemia but not in elderly mice. The study suggested that the protective effect of *rh*EPO on BBB integrity was age-dependent (Thériault et al., 2016). In addition, *rh*EPO treatment also restored the local cerebral blood flow in the penumbra and promoted angiogenesis and neurogenesis at the delayed stage of cerebral ischemia. It was reported that exogenous *rh*EPO could enhance the EPO level in the brain, increase the vascular endothelial growth factor (VEGF), and its receptor vascular endothelial growth factor receptor-2 expression and regulate HIF-1α and the endothelial nitric oxide synthase (eNOS) expression through activating AMPK-KLF2 signaling, eventually promoting angiogenesis at 7 days after cerebral ischemia (Chen GH. et al., 2019). In a mouse model of permanent MCAO, systemically applying *rh*EPO enhanced survival and proliferation of endothelial cells and upregulated several angiogenic factors at 14 days after ischemia (Li et al., 2007b). In an embolic stroke model in mice, increased angiogenesis and neurogenesis after *rh*EPO treatment were associated with the induction of vascular endothelial growth factor and brain-derived neurotrophic factor (BDNF) expressions along the infarct boundary 4 weeks after ischemia (Wang L. et al., 2004) Figure 4.

Recently, it has been found that *rh*EPO could suppress the activation of astrocytes and reduce the number of M1 microglia to promote angiogenesis and neurogenesis after cerebral ischemia (Zhang et al., 2019). Furthermore, through attenuating microglia activation and modulating microglia polarization, *rh*EPO could suppress neuroinflammation under pathological conditions (Bond and Rex, 2014). In a focal cerebral ischemia model in mice deficient for TGF-β-activated kinase 1 (TAK1) in microglia/macrophages, a study demonstrated that EPO administration improved clinical outcomes and dampened stroke-induced activation of TAK1 and inflammasome cascades (Heinisch et al., 2021). The neuroprotective effects were not evident after deleting Mi/MΦ TAK1. The study suggested that EPO could affect the EPO/TAK1/inflammasome axis to convey neuroprotection (Heinisch et al., 2021).

In a mouse model of subarachnoid hemorrhage, *rh*EPO promoted the polarization of M1 microglia toward the M2 phenotype and alleviated inflammation, partially through the EPOR/JAK2/STAT3 pathway (Wei et al., 2017). In a rat model of peripheral neuropathy, *rh*EPO could decrease microglial activation, diminish the release of pro-inflammatory cytokines, and reduce neuropathic pain behavior, which relied on the EPO receptor expressed on Schwann cells (Huang et al., 2018). After traumatic brain injury (TBI), *rh*EPO could reduce immune/inflammatory cell infiltration in the brain 3 days after injury, which was associated with reduced brain edema and improved cognitive function (Mitkovski et al., 2015; Zhou et al., 2017). However, to date, only a few studies investigated whether *rh*EPO modulated microglia activation and polarization after ischemic stroke to protect neurons, promote tissue regeneration, and functional recovery. In adult mice subjected to 45-min MCAO surgery, EPO injection reduced M1 microglia and increased M2 microglia at 14 days after ischemia in the brain, contributing to ameliorated white matter injury and improved neurobehavioral outcomes (Wang et al., 2017a). Further study demonstrated that a mutant EPO (MEPO) shifted microglia toward M2 polarization by promoting JAK2/STAT3 activation and inhibiting the expression of C/EBPβ at 14 days after cerebral ischemia-reperfusion in middle-aged (9-month-old) mice (Wang et al., 2021). These studies suggested that EPO and its derivatives have potentials to maintain the M2 microglia phenotype to accelerate white matter repair and improve outcomes after cerebral ischemia. However, the exact mechanism on how EPO affects microglia polarization toward the M2 phenotype needs further investigation.

#### 3.1.3 The Effect of EPO on Cognition in Ischemic Stroke

Emerging evidence proved that *rh*EPO increased the number of oligodendrocytes, attenuated axonal injury, and maintained white matter integrity under normal and ischemic conditions (Hassouna et al., 2016; Wang et al., 2017b). Enhanced axonal density and white matter integrity participated in attenuating the cognitive deficit after cerebral ischemia (Ma et al., 2015; Wang Y. et al., 2016; Wang et al., 2017a; Yu et al., 2019). Improved cognitive function was found after *rh*EPO treatment in animal models of hypoxic-ischemic encephalopathy (HIE), TBI, diabetics, abdominal surgery, electroconvulsive stimulation, psychiatric disorders and neonatal stroke (Lee et al., 2017; Othman et al., 2018; Schober et al., 2018; Razak and Hussain., 2019; Kjær et al., 2020), and in chronic kidney disease and mood disorder (Miskowiak et al., 2019; Vinothkumar et al., 2019).

Using a transient MCAO model in postnatal 10 (P10) Sprague–Dawley rats, a study demonstrated that multidose systemic EPO (1000 U/kg per dose×3 every 72 h) when administered at 3 or 7 days after ischemia enhanced long-term recognition memory and exploratory behavior in rats at 2 months of ages (Larpthaveesarp et al., 2021). In an early postnatal hyperoxia model, EPO had pro-myelinating effects and improved cognition in adolescent and adult rats (Dewan et al., 2020). In a model of germinal matrix-intraventricular hemorrhage (GM-IVH) of the preterm infant, EPO restored the neuronal density, ameliorated dendritic spine loss, and reduced inflammation and small vessel bleeding, contributing to the preservation of learning and memory abilities (Hierro-Bujalance et al., 2020). Recently, using a constitutively expressing transgenic mouse line, a study demonstrated that EPO stimulated the hippocampal-specific neuronal maturation and synaptogenesis early in postnatal development in mice, contributing to improved cognitive behaviors (Khalid et al., 2021). EPO’s acute and extended synaptic plasticity effects and its ability to modulate both excitatory and inhibitory neurotransmissions could partly contribute to the observed cognitive behavioral effects (Khalid et al., 2021). Further study proved that promoting mitochondrial function throughout early postnatal development also corresponded to enhanced cognition by early adulthood in mice (Jacobs et al., 2021). It was shown that EPOR exerted the central role in maturation of GABAergic interneurons in the hippocampus and EPO-driven neurogenesis (Wakhloo et al., 2020; Khalid et al., 2021). The aforementioned studies investigated the action of EPO on cognitive improvement in animals of neonatal stroke, hyperoxia, or hypoxia and during development. However, studies focusing on whether *rh*EPO improves the cognitive function in patients suffering from chronic brain ischemia or at the delayed stage of ischemic stroke are still lacking. Elucidating these questions could expand therapeutic ranges of EPO and help develop a novel therapy to improve the cognitive function.

### 3.2 EPO Treatment in the Human Clinical Trials of Ischemic Stroke

Over the past 3 decades, much work has been carried out to further characterize the therapeutic potential of *rh*EPO not only in experimental studies in rodents but also in patients with ischemic stroke (Table 1).

**TABLE 1:** Clinical trials of *rh*EPO treatment for ischemic stroke patients.

Trails	Patients	Number	Drug	Dose	Route	Time of treatment	Time of evaluation	Observations	Results	References
An exploratory double-blind study	Chronic stroke Patients (3 months after onset)	In a pilot study (*n* = 3); in an exploratory double-blind study (*n* = 6)	*rh*EPO + recombinant human G-CSF	*rh*EPO (300 U/kg); G-CSF (10 μg/kg)	Subcutaneous	Once a day for 5 days per month over 3 months	0, 5, and 30 days in each cycle and on day 180	Vital signs, adverse events, hematological values, and functional outcomes	No observations of serious adverse events. The grip power of the dominant hand was increased; mini-mental status examination (MMSE) and modified Barthel index (MBI) were not improved	Shin and Cho (2016)
A prospective, randomized, placebo-controlled trial (ISRCTN71371114)	Acute ischemic stroke who were not candidates for rtPA therapy at a single facility	EPO-treated group (*n* = 71); placebo-control group (n = 71)	*rh*EPO	5000 IU/dose	Subcutaneous	48 and 72 h after stroke	90 days	Recurrent stroke or death; long-term functional recovery (that is, 5 years)	Did not affect long-term recurrent stroke and mortality but reduced the scale of Barthel index; EPO therapy significantly improved long-term neurological outcomes	Tsai et al. (2015)
A randomized clinical trial	First acute ischemic stroke within 24 h of symptom onset	EPO-treated group (*n* = 37); placebo-control group (*n* = 43)	*rh*EPO	16,000 IU as a bolus dose and continued as 8000 IU each 12 h up to a total dose of 56,000 IU during 3 days	Iv	3 days after stroke	14 and 28 d	NIHSS	High dose of erythropoietin in first 24 h can be effective on reduction of ischemic stroke complication	Asadi et al. (2013)
Subgroup analysis from the data of the German Multicenter EPO Stroke Trial (Phase II/III; ClinicalTrials.gov Identifier: NCT00604630)	Acute ischemic stroke	*n* = 163	*rh*EPO; rtPA	40,000 IU each	Iv	Within 6 h of symptom onset, and at 24 and 48 h after stroke	1, 2, 3, 4 and 7 days	Serum biomarker profiles (S100b, GFAP, and ubiquitin C-terminal hydrolase (UCH-L1))	The reduction of serum biomarkers corroborated an advantageous effect of EPO in ischemic stroke	Ehrenreich et al. (2011)
Double-blind, placebo-controlled, randomized German Multicenter EPO Stroke Trial (Phase II/III; ClinicalTrials.gov Identifier: NCT00604630)	Acute ischemic stroke in the middle cerebral artery territory	*n* = 460	*rh*EPO; rtPA	40,000 IU each	Iv	Within 6 h of symptom onset, at 24 and 48 h after stroke	90 days	Primary outcome: Barthel Index	The treatment of rtPA and *rh*EPO did not show any improvement in clinical outcomes but had a higher overall death rate	Ehrenreich et al. (2009)

In 2002, Ehrenreich et al. reported that *rh*EPO given (iv, 3.3 × 10^4^ IU/50 ml/30 min) once daily for the first 3 days after ischemic stroke in 53 patients could penetrate to the MCA territory; the EPO level in the CSF could increase to 60–100 times compared to the untreated patients (Ehrenreich et al., 2002). In addition, administration of *rh*EPO given within 5 h of onset of symptoms was associated with an improvement in the follow-up and outcome scales 30 days’ post-stroke (Ehrenreich et al., 2002).

In 2009, Ehrenreich et al. showed the results of a double-blind, placebo-controlled, randomized German Multicenter EPO Stroke Trial of *rh*EPO treatment for acute ischemic stroke. The study enrolled 522 patients with acute ischemic stroke in the MCA territory with 460 patients treated as planned (per-protocol population). EPO was given (40,000 IU in each patient) within 6 h of symptom onset and repeated at 24 and 48 h. Unexpectedly, patients receiving both the recombinant tissue plasminogen activator (rtPA) and *rh*EPO treatment did not show the improvement of clinical outcomes but had a higher overall death rate 90 days after stroke (Ehrenreich et al., 2009). With the subgroup analysis, patients only receiving *rh*EPO benefitted from *rh*EPO. It was further corroborated by findings of lower concentrations of serum biomarker profiles including the glial markers of S100 calcium-binding protein B (S100B) and glial fibrillary acid protein (GFAP), the neuronal marker ubiquitin C-terminal hydrolase (UCH-L1), as an outcome measure of brain damage, in the serum of *rh*EPO-treated patients at 7 days of observation after symptom onset (Ehrenreich et al., 2011).

An experimental study was conducted in embolic MCAO rats; *rh*EPO (5,000 U/kg) in combination with rtPA (10 mg/kg) was treated at 2 or 6 h after MCAO. The results showed that *rh*EPO exacerbated rtPA-induced brain hemorrhage without reduction of ischemic brain damage when administered at 6 h. However, when the treatment initiated 2 h after MCAO, beneficial outcomes were achieved. The detrimental effects caused by delayed treatment of *rh*EPO combined with rtPA were associated with the increase of MMP-9, NF-kB, and IL-1 receptor-associated kinase-1 in the brain (Jia et al., 2010).

Several clinical trials conducted later by other groups obtained positive results. In 2013, a randomized clinical trial (37 patients of the *rh*EPO group and 43 of the control group) showed that administration of a high-dose *rh*EPO in first 24 h was effective on reduction of ischemic stroke complication. The patients enrolled were diagnosed with first ischemic stroke. The dose of the applied *rh*EPO was 16,000 IU as a bolus IV and continued as 8,000 IU per 12 h up to a total dose of 56,000 IU within 3 days. The evaluation time point was 14 and 28 days after the stroke attack (Asadi et al., 2013). With a 90 days follow–up, Tsai et al. (2015) showed that two consecutive doses of *rh*EPO (5,000 IU/dose, subcutaneously administered at 48 and 72 h after acute ischemic stroke, 71 patients of the *rh*EPO group) did not affect long-term recurrent stroke and mortality but reduced the Barthel index scale.

In 2016, another clinical study proved the combination therapeutic effects of EPO and granulocyte colony-stimulating factor in ischemic stroke patients. Different from the former studies focusing on acute ischemic stroke patients, the study enrolled nine chronic stroke patients, at least 3 months after ischemic or hemorrhagic stroke. The study reported that subcutaneous infusion of *rh*EPO (300 U/kg) in the sodium chloride solution, once a day for five consecutive days, showed no adverse effects and enhanced the grip power of the dominant hand at 6 months after stroke (Shin and Cho, 2016).

These aforementioned clinical studies suggested that the *rh*EPO was safe and effective for improving long-term neurological function. However, a recent systematic review and meta-analysis, which contained four randomized controlled trials involving 784 patients to elucidate the role of *rh*EPO in treating patients with acute ischemic stroke, made a different conclusion. The authors defined 30-day National Institutes of Health Stroke Scale (NIHSS) measures as a primary outcome and the 90-day Barthel Index as a secondary outcome. As a conclusion, they did not recommend *rh*EPO for patients with acute ischemic stroke, especially with the combination of rtPA (Yao et al., 2017).

Although experimental studies showed the wide range of the *rh*EPO usage, clinical studies of *rh*EPO for ischemic stroke therapy, especially in the chronic stage, were still uncommon. Considering the low efficiency of clinical translation of *rh*EPO, pre-clinical studies need to be planned very carefully in the future. Factors like age and comorbidities may compromise the therapeutic effects, which should be considered in the design of the experimental study for exploring the neurotherapeutic potential of *rh*EPO in ischemic brain injury (Simon et al., 2019). When it comes to clinical trials, large-scale randomized controlled trials, evaluating the beneficial effects of *rh*EPO given within 6 h after symptom onset, were needed for acute ischemic stroke patients. Based on the previous clinical studies, patients presenting with NHISS>=4 and MCAO should be included (Ehrenreich et al., 2009; Ehrenreich et al., 2011). When *rh*EPO was given at a delayed time of 48 h after symptom onset, the inclusion criteria included a scoring of >2 on the NIHSS and a time window of <=48 h from the onset of symptoms (Tsai et al., 2015). Furthermore, large-scale randomized controlled trials were also needed to further explore the therapeutic effects of *rh*EPO on chronic ischemic or hemorrhagic stroke patients at least 3 months after symptom onset (Shin and Cho., 2016).

## 4 EPO Treatment in Pediatric Stroke and Neonatal Encephalopathy

Apart from adult stroke, the efficacy of EPO treatment was evaluated in pediatric stroke and neonatal encephalopathy. In a neonatal stroke model of MCAO in P10 rats, EPO treatment (5 U/g, ip) preserved hemispheric brain volume 6 weeks after injury. Furthermore, EPO increased the percentage of newly generated neurons while decreased newly generated astrocytes following brain injury. The study proved that EPO enhanced long-term neuroprotection and neurogenesis in neonatal stroke (Gonzalez et al., 2007). In clinical practice, several clinical trials reported that human infants with HIE who received multiple doses of EPO during the first week of age, in the absence of or combined with hypothermia, experienced improved neurological outcomes (Elmahdy et al., 2010; Wu et al., 2012). For a longer-term outcome, Elizabeth E et al. firstly described the neurodevelopmental outcomes in infants who received six doses of EPO with hypothermia during the neonatal period. They provided evidence that high-dose EPO given in conjunction with hypothermia to treat newborns with HIE did not worsen outcomes at median age 22 months (Rogers et al., 2014). However, this was a small, open-labeled study with no controls. Future study with a blinding and consistent length and quality of follow-up is warranted to assess the efficacy of EPO treatment in conjunction with hypothermia for HIE infants.

Recently, a recent systematic review and meta-analysis identified the positive effects of EPO (1,500–12,500 UI/kg/dose) or a derivative monotherapy on near-term and term infants with neonatal encephalopathy. The data showed that EPO administrated after the birth of infants reduced the risk of death (during the neonatal period and at follow-up) or neuro-disability at 18 months or later (Ivain et al., 2021). However, the study only retrieved five studies in low-to-middle income countries, which made it a poor evidence. Clinical studies clarifying the therapeutic effects of EPO on mature infants are needed in the future.

## 5 EPO Treatment in Premature Infants

Recently, the Preterm Erythropoietin Neuroprotection (PENUT) Trial measured the plasma potential biomarkers of neurological injury in extremely preterm (<28 weeks’ gestation) infants. The trail aimed to determine whether biomarkers of hypoxia and inflammation were associated with outcomes at two years of age and whether EPO treatment decreased markers of inflammation (*n* =391 EPO, *n* =384 placebo). The trail found that elevated baseline EPO (within 24 h after birth before the first study drug administration) was associated with increased risk of death or severe disability of the infants at two years of age, while EPO when administrated after the birth did not decrease markers of inflammation or affect outcomes at any treatment time (Wood et al., 2021). Further study performed by the same group confirmed that there was no effect on long-term neurodevelopment in EPO-treated extremely preterm infants even in the presence of microstructural changes identified by MRI with diffusion tensor imaging (DTI) (Law et al., 2021). Previous studies demonstrated that preterm birth was often associated with perinatal insults such as growth restriction, hypoxia, and ischemia (Galinsky et al., 2013). During chronic fetal hypoxia or as a part of inflammatory response, endogenous EPO was induced to protect the brain and other vital organs (Logan et al., 2014; Teramo et al., 2018). Therefore, the elevated endogenous EPO possibly exerted neuroprotective functions as a response to hypoxia and was a potential biomarker of prolonged in utero hypoxia. However, based on the negative results of the Preterm Erythropoietin Neuroprotection Trial, the neuroprotective effects of exogenous EPO on premature infants after birth need further investigation.

## 6 EPO Treatment In TBI

Apart from acute stroke, EPO was used to treat TBI. Therapeutic effects of EPO included improving post-traumatic cerebral blood flow, pressure autoregulation, and vascular reactivity to l-arginine. These cerebral hemodynamic effects of EPO partly depended on nitric oxide generated by endothelial nitric oxide synthase (Cruz Navarro et al., 2014). EPO administration prior to or within few hours or even 1 day after TBI could enhance neurogenesis and improve functional outcomes in rats (Lu et al., 2005; Peng et al., 2014). EPO administration could also attenuate motor and cognitive deficits in TBI rats, possibly associated with upregulating the EPO receptor and reducing CD68^+^ cells (Hellewell et al., 2013). Studies indicated that the neuroprotective capacity was only bolstered under hypoxic conditions, which was an important consideration when EPO was employed for neuroprotection in the clinic. Recent studies showed that EPO was able to reduce brain edema at 1 and 4 days after TBI (Blixt et al., 2018). The neuroprotective function was elicited by increasing the mRNA brain-derived neurotrophic factor expression and serum SDF-1 levels within 24 h after TBI in rats (Said et al., 2021). In addition, a novel strategy was developed to load EPO with Tween 80-modified albumin nanoparticles using electrostatic spray technology. The results showed that the loaded EPO enhanced the distribution of EPO in the brain and relieved brain edema more effectively in TBI rats (Xue et al., 2020). These studies indicated that EPO held huge therapeutic potential in reducing brain edema in TBI patients.

In terms of clinical trials, the efficacy of EPO in patients with TBI yielded conflicting results. In 2010, the first randomized trial of EPO in TBI patients included 11 patients in the EPO group and five patients in the placebo group. The results showed that a dose of 40,000 units of EPO within 6 h of injury did not reduce neuronal cell death compared to placebo. TBI severity was worse in the EPO group. The outcomes of death, length of stay, and Glasgow outcome scores were also not affected after EPO treatment. The authors speculated that a larger trial with an ideal therapeutic dose might help determine if EPO was neuroprotective for TBI patients (Nirula et al., 2010). Five years later, a double-blind, placebo-controlled trial enrolling larger size patients of TBI (*n* = 606) was undertaken in 29 centers in seven countries. The study found that following moderate or severe TBI, EPO administration within 24 h of brain injury (40,000 units subcutaneously, per week for a maximum of three doses) did not improve neurological outcomes or increase the incidence of deep venous thrombosis of the lower limbs (Nichol et al., 2015). Further analysis adjusting for TBI severity showed that six-month mortality was lower in EPO-treated patients (Skrifvars et al., 2018). The subgroup of TBI patients with a diffuse type of injury not requiring a neurosurgical intervention prior to randomization might be benefitted from EPO treatment, while neuronal and axonal markers and glial biomarker concentrations in the serum were not affected by EPO (Hellewell et al., 2020).

Recently, studies of meta-analysis assessed the effectiveness of EPO on mortality, neurological outcomes, and adverse events in the treatment of TBI patients. The studies did not demonstrate a beneficial effect of EPO intervention on neurological recovery, hospital mortality, and risk of deep vein thrombosis (Liu M. et al., 2020; Liu C. et al., 2020; Katiyar et al., 2020). However, secondary analysis revealed that EPO reduced 6-month mortality in TBI patients. The authors found that the follow-up duration and the severity of injury had an impact on the stability of the results (Benoit et al., 2019; Katiyar et al., 2020). Large trials with consideration on timing of measurement and injury severity are warranted to evaluate the role of EPO in patients with TBI.

## 7 EPO Derivative Treatment for Ischemic Stroke

Despite the fact that exogenous EPO could cross BBB, the level of EPO penetrating into the brain was low during systematic injection. In addition, the affinity of EPORs expressed in non-hematopoietic tissues to EPO was not higher. Therefore, a high dose of exogenous EPO was needed to allow it to cross the BBB into brain tissues and achieve effective therapeutic concentrations in neurological diseases. The effective dose of EPO for humans with stroke (70 kg body weight, 33,000 U daily) was much higher than that of chronic kidney disease or anemia (70 kg body weight, 1,050–3,500 U daily) (Wang R. et al., 2016). Several clinical studies showed that EPO with a concentration of 50–150 U/kg daily could markedly increase the risk of thrombosis and brain injury (Beguin, 1999; Stohlawetz et al., 2000; Ehrenreich et al., 2009; Sargin et al., 2010). To exacerbate, high doses of EPO could cause other unexpected side effects, such as secondary infarction, seizures, thrombus development in arterio-venous shunt, and hypertension, which could further induce encephalopathy and seizures (Wolf et al., 1997; Brines et al., 2000; Agarwal, 2018; Tatlı et al., 2020). To avoid side effects, a variety of EPO derivatives including modified EPO molecules and peptides that mimic the 3D structure of EPO have been developed (Brines et al., 2008). EPO derivatives have shown therapeutic effects with low or without erythropoiesis effects in cerebral ischemia models (Table 2).

**TABLE 2:** Characteristics of different derivatives of *rh*EPO. Asialoerythropoietin (Asialo-EPO); carbamylated EPO (CEPO); neuro-EPO; EPOL; Darbepoetin alfa.

Derivatives	Source	Structure	Hematocrit Effects	Therapeutic effects	Doses	Routes	Mechanisms	Model or clinical trials	Half-time	References
*rh*EPO	Chinese hamster ovary cells	Composed of 166 amino acids, with a globular three-dimensional structure of four amphipathic α helices, two β-sheets, and two intra-chain disulfide bridges	High	Neuroprotective effects and improve cognitive function	500–5000IU/kg in rodents; 5000–4000 IU/dose in patients	Ip, iv, intranasal or subcutaneous or intra-artery injection	Anti-inflammation, anti-apoptosis, angiogenesis, neurogenesis, immunoregulation etc.	Multiple acute brain injuries; neurodegenerative disorders; psychiatric disorders et al	8.5 h	Ibbotson and Goa (2001), Cantarelli et al. (2019), Rey et al. (2019), Jarero-Basulto et al. (2020), Peng et al. (2020), Newton and Sathyanesan (2021)
asialo-EPO	From genetically engineered tobacco plants	Deglycosylated form of EPO	Low	Neuroprotective, cardioprotective, and renoprotective effects	44 μg/kg bw; 80 ng/g	Ip, iv	Inhibited caspase-3/-9 activation; reduced mitophagy and autophagy markers	MCAO	1.4 min	Price et al. (2010), Kittur et al. (2015), Peng et al. (2020), He et al. (2021)
CEPO	A chemically modified derivative of EPO	Replace lysines with homocitrulines	Low	Neuroprotective functions	50 μg/kg		Suppressed the expression of pro-apoptotic protein CC3 in the brain and regulated the Bcl-2/Bax ratio; protected neurons from ischemia through the CD131/GDNF/AKT pathway	MCAO		Villa et al. (2007), Wang et al. (2007), Oh et al. (2012), Ding et al. (2017)
Neuro-EPO	Chinese hamster ovary cells	Low sialic acid content in structure, rapidly degraded in the liver, and must be delivered by an intranasal route	None	Neuroprotective effects and improve cognitive function	249 UI/10 μl for animals; 100 ng/ml for cells; 0.5 or 1 mg for patients	Intranasal injection	Upregulated Bcl-2 and inhibited glutamate-induced caspase-3 activation	Cerebral ischemia, AD, and PD		Teste et al. (2012), Rodríguez Cruz et al. (2017), Fernando et al. (2018), Garzón et al. (2018), Pedroso et al. (2018), Rama et al. (2019), García-Llano et al. (2021)
EPOL	Isolated from skimmed goat milk	A low salivated bi-antennary structure	None	Neuroprotective effect	88 μg/kg for mice; at least 1 ng/ml or 10 ng/ml when treating cultured cells	Iv	Activated intracellular JAK/STAT and upregulated Bcl-2	Oxidative stress and AD model *in vitro*		Castillo et al. (2018), Castillo et al. (2019)
Darbepoetin alfa	A hyperglycosylated *rh*EPO analog	Additional sialic acid-containing oligosaccharide chains	Higher than *rh*EPO	Neuroprotective effects and improve cognitive function	10 mg/kg or 5,000 U/kg for rats; 1 μg/kg or 4 μg/kg for infants; 10 μg/kg for preterm infants; 500 μg for anemia patients every 3 weeks for 24 weeks	Ip, iv, subcutaneous injection		MCAO, four-vessel occlusion, intracerebral hemorrhage, and infants or preterm infants	3-fold longer than that of *rh*EPO	Egrie et al. (2003), Belayev et al. (2005), Grasso et al. (2009), Samson et al. (2010), Patel and Ohls (2015), Ohls et al. (2016), Ohlsson and Aher (2017), Platzbecker et al. (2017)

### 7.1 EPOL

EPOL is a new variant of recombinant EPO expressed in the mammary gland tissue (Toledo et al., 2006). EPOL has a different glycosylation pattern with a low salivated bi-antennary structure and was isolated from skimmed goat milk (Castillo et al., 2018). A study using an ischemia model showed that EPOL did not cause hematopoietic activity but prevented neurons and the slice of the hippocampus against oxidative stress through activating its receptor. The intracellular JAK/STAT activation and Bcl-2 gene upregulation were involved in neuroprotection (Castillo et al., 2018). Another study from the same group showed that EPOL could combat against Aβ-induced oxidative stress by a mechanism mediated by the EPO receptor. It has been reported that the effective concentration of EPOL was 10 times lower than that of *rh*EPO (Castillo et al., 2019). These studies indicated that EPOL represented a potential biopharmaceutical candidate to treat different CNS diseases.

### 7.2 Neuro-EPO

Neuro-EPO is a recombinant human glycoprotein produced in Chinese hamster ovary cells and can be obtained from the Center of Molecular Immunology (CIM, Havana, Cuba) (Rama et al., 2019). Different from the structure of EPO synthesized in kidney, neuro-EPO is characterized by its low sialic acid content, which is less than 10 per mole of EPO (Garcia Rodriguez and Sosa Teste, 2009). Because neuro-EPO is rapidly degraded in the liver, it must be preferentially delivered by an intranasal route, which is devoid of inducing the EPO activity (Garcia Rodriguez and Sosa Teste, 2009; Rama et al., 2019). Neuro-EPO is not chemically modified and biologically similar to endogenous EPO synthesized in the mammalian brain, which is referred to as a neuro-EPO (Garcia Rodriguez and Sosa Teste, 2009; Merelli et al., 2018).

Neuro-EPO was demonstrated to exert neuroprotective effects in models of cerebral ischemia both *in vitro* and *in vivo* (Rodríguez Cruz et al., 2010; Cesar Garcia-Rodriguez and Rodriguez-Cruz, 2012; Teste et al., 2012). In a Mongolian gerbil model of right common carotid artery (CCA) ligation, the average dose of used neuro-EPO (249 UI/10 μl/every 8 h for 4 days) showed a 25% higher viability efficacy than the control (Teste et al., 2012). The study concluded that neuro-EPO application starting within 12 h after ischemia improved neurological scores and behavior of the spontaneous exploratory activity after 7 days of ischemia (Teste et al., 2012). Using an *in vitro* model of cerebral ischemia, in which oxidative stress was induced by glutamate in cultured neurons, neuro-EPO (100 ng/ml) treatment preserved neurons from oxidative stress through upregulation of Bcl-2 and inhibition of glutamate-induced caspase-3 activation (Fernando et al., 2018; Garzón et al., 2018). In addition, neuro-EPO was used in AD models and showed improvement in cognitive function (Maurice et al., 2013; Rodríguez Cruz et al., 2017). Recently, a phase I clinical trial demonstrated that 0.5 or 1 mg of neuro-EPO delivered intranasally every 8 h in 4 days was safe and did not cause severe adverse events (Santos-Morales et al., 2017). Furthermore, neuro-EPO improved cognitive functions in PD patients. Another clinical trial phase III with neuro-EPO treatment for PD patients is in progress to confirm its neuroprotective properties (Pedroso et al., 2018). The preliminary results showed that nasally administered neuro-EPO for 5 weeks in patients with PD stages 1 and 2 on the Hoehn & Yahr Scale was well-tolerated (García-Llano et al., 2021).

Currently, no effective methodology is available to activate endogenous EPO production by the brain. Therefore, neuro-EPO is an attractive candidate for neuroprotective therapy because its biological activity is similar to endogenous EPO synthesized in the mammalian brain. Additionally, neuro-EPO elicited positive effects not only on neurons but also on glia cells (Merelli et al., 2018; Rama et al., 2019). More pre-clinical and clinical studies are warranted to explore the safety and efficiency of neuro-EPO in acute and chronic ischemic stroke.

### 7.3 CEPO

CEPO is a chemically modified derivative of EPO and has been shown to promote hippocampal neurogenesis and neuronal differentiation in adult mice but not affect neurogenesis in the developing rat brain under normal conditions (Oh et al., 2012; Osato et al., 2018). After irradiation to the developing rat brain, CEPO even attenuated neurogenesis in the sub-ventricular zone (SVZ) (Osato et al., 2018). It was reported that CEPO did not bind to the classical EPOR *in vitro* or elicited a hematopoietic response *in vivo* but exerted neuroprotective functions after treatment in animal models of cerebral ischemia or other types of neuronal injury (Leist et al., 2004). In cultured primary neurons under oxygen-glucose deprivation (OGD) and mice with hypoxia-re-oxygenation, CEPO promoted neurogenesis and showed neuroprotective effects. The blockage of CD131 (βcR), a subunit of the EPO receptor (EPOR/βcR), reduced CEPO-mediated glial-derived neurotrophic factor (GDNF) production. GFR receptor blockage and GDNF neutralization inhibited CEPO-induced neurogenesis. Thus, the study indicated that CEPO protected neurons from ischemia possibly through the CD131/GDNF/AKT pathway (Ding et al., 2017).

In a rat model of embolic MCAO, CEPO treatment (50 μg/kg, at 6, 24, and 48 h after MCAO) reduced the cerebral infarct volume, the number of apoptotic cells, and activated microglia in the ischemic boundary region, facilitating neurological functional recovery at 28 days after MCAO (Wang et al., 2007). Another study confirmed the neuroprotective effects of CEPO treatment (50 μg/kg) in cerebral ischemia. CEPO treatment inhibited neuroinflammation in the brain of rats from 1 to 60 days postoperatively. Even if applied 24 h after ischemia, improved functional recovery also was observed (Villa et al., 2007). These studies suggested that CEPO treatment exerted beneficial functions both at the acute stage and the long-term recovery phase of cerebral ischemia. In a fetal rat model of HIE, CEPO treatment suppressed the expression of pro-apoptotic protein cleaved caspase-3 (CC3) in the brain and regulated the Bcl-2/Bax ratio, resulting in reduced neuronal apoptosis (Diao et al., 2019). Beyond cerebral ischemia, CEPO exhibited neuroprotective functions in other neurological diseases including AD (Hooshmandi et al., 2018; Hooshmandi et al., 2020; Moosavi et al., 2020), PD (Thomas Tayra et al., 2013), periventricular leukomalacia (Liu et al., 2011), TBI (Millet et al., 2016), spinal cord depression and hemisection (King et al., 2007), and sciatic nerve compression (Chen et al., 2015).

However, studies focusing on CEPO therapy for ischemic stroke patients in clinics are still lacking. In addition, the cellular and molecular mechanism of CEPO treatment needs to be explored further in pre-clinical studies. Using the mass spectrometry-based proteomics, a recent study compared EPO and CEPO-induced protein profiles in neuronal phenotype PC12 cells. The bioinformatics enrichment analysis showed that EPO and CEPO induced different protein expressions in different regions of the brain (Tiwari et al., 2021). For example, synaptic plasticity-related protein cortactin was induced by CEPO in the molecular layer, while pleiotrophin was increased in the vasculature by EPO in the rat brain (Tiwari et al., 2021). The study shed light on potential mechanisms of EPO- and CEPO-produced cognitive-enhancing effects in pre-clinical studies. Future studies investigating the mechanism of CEPO treatment help accelerate the speed of clinical translation of CEPO in neurological diseases.

### 7.4 Asialo-Epo

Asialo-EPO is a desialylated form of human EPO and could be produced and purified from genetically engineered tobacco plants (Kittur et al., 2015). It is noted that the sialic acids at the end of the oligosaccharide chains of EPO is helpful for maintaining its stability *in vivo*. Therefore, the desalination of EPO made asialo-EPO a short half-life (only 1.4 min), which had an insufficient time to stimulate hematopoiesis but still displayed complete neuroprotection (Chen et al., 2015; Peng et al., 2020). Asialo-EPO was reported to exert neuroprotective, cardioprotective, and renoprotective effects in organ injuries including cerebral ischemia (Hermann, 2009; Chen et al., 2015; Kittur et al., 2015). In a rat transient MCAO model, a single or repeat administrations of asialo-EPO-liposomes immediately after reperfusion ameliorated ischemic brain injury and neurological deficit after 7 days of injection (Ishii et al., 2012a; Ishii et al., 2012b). Asialo-EPO could be detected in the CSF when it was continuously infused (Price et al., 2010). These studies demonstrated that asialo-EPO provided at least short-term neuroprotective effects after cerebral ischemia, associated with inhibiting caspase-3/-9 activation and reducing the number of apoptotic neurons (Price et al., 2010).

In a neonatal hypoxia/ischemia model in 7-day-old rats, asialo-EPO (80 ng/g) injected IP 4 h before ischemia reduced the cerebral infarct volume at 5 days post-surgery. The protective function was related to reduction of ERK phosphorylation and upregulation of the synaptosome-associated protein of 25 kDa (SNAP-25) (Wang X. et al., 2004). Recently, a study demonstrated that asialo-EPO treatment by repeated intravenous injection (44 μg/kg bw) in mice showed neuroprotective effects in a cerebral ischemia and reperfusion (I/R) mouse model. The therapeutic mechanism included preventing ischemia and reperfusion injury-induced increase in mitophagy and autophagy markers and inhibiting apoptosis to benefit nerve cell survival. Furthermore, the study found that asialo-EPO did not cause erythropoietic activity and immunogenicity, which held great translational potential as a multimodal neuroprotective drug for stroke treatment (He et al., 2021). Overall, asialo-EPO therapy showed positive effects. However, the study of safety, advantages, and molecular mechanisms of asialo-EPO therapy is insufficient. Thus, further investigation is still needed to elucidate these questions to make it applicable for clinics.

### 7.5 Darbepoetin Alfa

Darbepoetin alfa is a novel erythropoiesis-stimulating agent with two additional N-glycosylation sites and up to 22 sialic acid moieties, extending its circulating half-life 3-fold longer than that of *rh*EPO (Egrie et al., 2003). In addition, darbepoetin alfa had a higher bioactivity in increasing the hematocrit in normal mice (Egrie et al., 2003). Pre-clinical studies demonstrated that darbepoetin alfa treatment exerted neuroprotective effects in several models of neuronal injury including focal or global cerebral ischemia and experimental intracerebral hemorrhage (Belayev et al., 2005; Grasso et al., 2009; Samson et al., 2010). However, it is possibly due to the side effects of erythropoiesis-stimulation, clinical trials exploring the therapeutic effect of darbepoetin alfa for ischemic or intracerebral brain injury were absent. In contrast, many clinical trials demonstrated the erythropoietic and potential neuroprotective effects of darbepoetin alfa treatment in term infants, preterm or low birth weight infants (Patel and Ohls, 2015; Ohlsson and Aher, 2017). Recently, a phase III randomized placebo-controlled trial showed that darbepoetin alfa treatment (500 μg) was safe in patients with anemia and lower-risk myelodysplastic syndromes, contributing to reduced transfusion incidence and increased rates of erythroid response (Platzbecker et al., 2017). Although previous clinical trials had positive results, no new ongoing trials were identified, and no clinical studies used darbepoetin alfa in infants until now. Studies evaluating the therapeutic effect of darbepoetin alfa in patients with anemia and acute brain injury including cerebral ischemia are urgently needed.

### 7.6 Other Derivatives

Other derivatives included a group of peptides derived from EPO such as S104I-EPO (Gan et al., 2012), Epobis (Dmytriyeva et al., 2016), pyroglutamate helix B surface peptide (pHBSP; ARA-290; Collino et al., 2015; Zhang et al., 2017), JM4 (Wang B. et al., 2016), and S100E (Villa et al., 2007). These derivatives crossed BBB and showed neuroprotective effects without stimulating erythropoiesis in a wide range of neurological diseases (Brines and Cerami, 2005; Collino et al., 2015; Zhang et al., 2017; Kunze and Marti., 2019; Peng et al., 2020). Pre-clinical studies are still expected to demonstrate the safety and efficiency of these derivative therapies. Additionally, the specific binding site of EPORs and mechanism of these derivatives’ application in cerebral ischemia are not well known and need further investigation to expand the ischemic stroke therapeutic strategies.

## 8 Discussion

Increasing evidence has shown that EPO not only regulates erythropoiesis in response to hypoxia but also exerts non-hematopoietic effects such as anti-apoptosis, antioxidant, anti-inflammation, neuroprotection, angiogenesis, and immune regulation in a variety of non-hematopoietic tissues. It is noted that HIF gene upregulates the EPO expression under hypoxic conditions. HIF prolyl hydroxylase (HIF-PH) targeted HIF-α subunits and decreased the HIF activity (Muchnik and Kaplan, 2011). HIF-PH inhibitors have been demonstrated to stabilize the HIF and increase the HIF-dependent expression of EPO (Semenza, 2019). Increasing the HIF activity through HIF-PH inhibitors such as molidustat (BAY 85-3934) and roxadustat (FG-4592) were approved in phase III clinical trials on anemia in patients with chronic kidney disease (Beck et al., 2018; Chen N. et al., 2019; Semenza, 2019). After ischemic stroke, EPO and EPORs were upregulated and involved in protecting ischemic neurons and promoting tissue regeneration. However, endogenous EPO was insufficient to resolve ischemia-induced tissue injury. Previous studies reported that the selective small molecule inhibitor of HIF-PHs, 2-(1-chloro-4-hydroxyisoquinoline-3-carboxamido) acetic acid (IOX3), administered 24 h before MCAO contributed to neuroprotection partially because of BBB protection (Chen et al., 2014). Therefore, HIF-PH inhibitors hold a clinical use in elevating EPO levels and preventing damage related to ischemia-reperfusion such as cerebral ischemia, cardiac ischemia, and ischemic renal failure (Sulser et al., 2020).

Apart from enhancing the endogenous production level of EPO, the administration of exogenous *rh*EPO has been demonstrated to reduce ischemic brain injury and improve functional recovery both at the acute and late stages of cerebral ischemia in the pre-clinical and clinical studies. However, the molecular mechanisms underlying the neuroprotection of *rh*EPO are not yet fully understood. Furthermore, pre-clinical studies comparing the doses, routes, and times of EPO administration in ischemic stroke models are essential for its clinical translation. Finally, a larger clinical trial to evaluate the safety and efficiency of *rh*EPO treatment for ischemic stroke, especially chronic ischemic stroke, is urgent needed.

Recently, *rh*EPO with modification or combined with other therapies such as human umbilical cord blood cells, granulocyte colony-stimulating factor, and cyclosporine A displayed better beneficial effects than *rh*EPO treatment alone in rodent stroke models (Yu et al., 2014; Yuen et al., 2017; Hwang et al., 2019; Jeong et al., 2019; Zhang et al., 2019). Although exogenous EPO could penetrate into the ischemic brain, the concentration of EPO was low. It required a high dose and several infusion times to make EPO reach the concentration of effective therapy, which could result in thrombosis and cerebral injury. Intranasal delivery is a promising alternative route because it is easy and reliable to directly deliver the drugs into the brain. Compared to systemic administration, intranasal delivery allowed drugs to reach the brain rapidly, which reduced the risk of adverse effects. Several strategies such as loaded with nanoparticles (Jeong et al., 2019), fused to a chimeric monoclonal antibody targeting the transferrin receptor (Chang et al., 2018), and modified with liposomes increased EPO penetration into the brain (Fukuta et al., 2019). These novel technologies hold a potential for enhancing therapeutic effects of EPO and reducing its adverse effects. In addition, to eliminate the deleterious effects, a group of EPO derivatives were developed and showed neuroprotection in animal models of neurological disorders. CEPO, one of EPO derivatives, was proved safe in healthy volunteers and improved cognitive functions in PD patients, while it has not been studied in ischemic stroke patients. The safety and efficiency of EPO derivatives for ischemic stroke remains unclear and is warranted further exploration. The specific binding site of EPORs and the cellular and molecular mechanism of application of EPO derivatives are also not well understood. A thorough investigation of EPO derivatives is awaited to help yield enhanced understanding of their therapeutic mechanism and develop the potential novel therapeutic strategies for ischemic stroke.

## Abbreviations

AD, Alzheimer’s disease; asialo-EPO, asialoerythropoietin; BBB, blood–brain barrier; CEPO, carbamylated erythropoietin; CNS, central nervous system; CSF, cerebrospinal fluid; CRLF3, cytokine receptor-like factor 3; EPORs, EPO receptors; EPOR, EPOR/EPOR; EPO, erythropoietin; GDNF, glial-derived neurotrophic factor; HIF, hypoxia-inducible factor; HIE, hypoxic-ischemic encephalopathy; JAK, Janus kinase; MMP, matrix metalloproteinase; MCAO, middle cerebral artery occlusion; NIHSS, National Institutes of Health Stroke Scale; PD, Parkinson’s disease; PH, prolyl hydroxylase; *rh*EPO, recombinant human EPO; rtPA, recombinant tissue plasminogen activator; TBI, traumatic brain injury.
