# Supplementary material for: The Effect of Erythropoietin and Its Derivatives on Ischemic Stroke Therapy: A Comprehensive Review
Source: Front Pharmacol. 2022 Feb 17;13:743926. doi: 10.3389/fphar.2022.743926 (PMC8892214; doi:10.3389/fphar.2022.743926)
Supplement: Supplementary file 1 [file Table1.DOCX]

Table1. Pre-clinical studies of EPO treatment in ischemic stroke models.

| Animal | Model | Drug | Dose | Route | Time of injection | Time of observation | Outcome | Mechanism | Reference |
| --- | --- | --- | --- | --- | --- | --- | --- | --- | --- |
| C57 BL/6 mice (9-month-old) | MCAO 45min | MEPO | MEPO (5000  IU/kg) | Intraperitoneal | At the beginning of  reperfusion, 3, 5, 7, 9, 11d after ischemia | 14d | Improved neurobehavioral outcomes, alleviated brain tissue loss, and ameliorated white matter injury | Facilitated microglia toward the beneficial M2 phenotype to promote oligodendrogenesis via JAK2/STAT3 and  the C/EBPβ signaling pathway. | (Wang et al., 2021) |
| Postnatal day (P) 10 Sprague-Dawley rats | MCAO 180min | EPO or EPO+MSC or MSC | EPO (1000 u/kg per dose×3 every 72h) | Intravenous | 3, 7d after birth | 2 months | Improved recognition memory and exploratory behavior and reduced anxiety. | Not studied. | (Larpthaveesarp et al., 2021) |
| Male Wistar rats | MCAO 90min | EPO | EPO (5,000 IU/kg) | Intravenous | 15min prior to ischemia | 1d | Reduced infarct volume and midline shift | Increased regional CBF in cortical areas of the ischemic infarct. | (Juenemann et al., 2020) |
| Near-term fetal sheep | Global cerebral ischemia for 30min | EPO, EPO+ hypothermia | EPO (5000 U/kg loading dose, then 833.3 U/kg/h) | Intravenous | 3 to 72h post ischemia | 7d | Improved neuronal survival in the parasagittal cortex, hippocampal CA4 and thalamus | Reduced cortical caspase-3 and activated microglia in striatal and cortical areas. | (Wassink et al., 2020) |
| Rats | MCAO | EPO-CA-NPs （nanoparticles loaded with EPO） |  |  |  | 1, 3, 5, 7d | Reduced infarct volume and cellular apoptosis; improved sensorimotor functions | Not studied. | (Jeong et al., 2019) |
| C57BL/6 mice | MCAO 45min | MEPO (non-erythropoietic mutant erythropoietin); EPO | MEPO (5000 U/kg); EPO (5000 U/kg) | Intraperitoneal | Every other day until day 11 at the beginning of reperfusion | 1, 3, 7, 14, 21, and 28d | Increased survival rate, reduced brain tissue loss, and improved neurological function | Both EPO and MEPO promoted neurogenesis and angiogenesis but suppressed gliogenesis. | (Zhang et al., 2019) |
| Sprague Dawley rats | MCAO 1h | An EPO‐ and enhanced green fluorescence protein (EGFP)‐pro‐ ducing NIH/3T3 fibroblast cell line (EPO/EGFP/3T3) | 10X6 cells in 8 μl of PBS | Intracerebral injection at the right striatum | 2d after MCAO (day 1) | 3, 7, and 14d | Reduced infarct size and improved post‐ stroke functional recovery | Enhanced neural stem/progenitor cells proliferation and neuronal differentiation via the effect of EPO and BDNF secreted from fibroblasts. | (Chou et al., 2019) |
| Sprague-Dawley rats | MCAO 1.5h | HUCBC, EPO or HUCBC+EPO | HUCBC (1.2 × 10^7^); EPO (500 IU/kg) | HUVEC (tail vein); EPO (intraperitoneal) | For five consecutive days from 7d after MCAO | 28d | Improved neurological function; HUCBC+EPO achieved the best effect. | Enhanced neurogenesis and angiogenesis, and reduced astrogliosis. | (Hwang et al., 2019) |
| C57BL/6 mice | Permanent MCAO | EPO | 5000 IU/kg | Intraperitoneal | Immediately after reperfusion and the following day | 7d | Improved survival rate and neurological function. | Promoted VEGF and its receptor (KDR) expression and regulated HIF-1α and eNOS protein expression through the activation of AMPK-KLF2 signaling pathways to promote new vascular development. | (Chen et al., 2019a) |
| Grina^-/-^ and wild type mice | MCAO 30min | EPO | 5000 U/kg | Intraperitoneal | 0, 24 and 48h after MCAO | 6 and 72h | Grina^-/-^ mice showed earlier and larger brain infarct volume and worse neurological outcome, and abolished EPO-mediated neuroprotection. | Enhanced the post-ischemic activation of pro-survival IRE1a and counteracted the pro-apoptotic PERK branch of the UPR (unfolded protein response) | (Habib et al., 2019a) |
| GRINA^-/-^, FAIM2^-/-^, GRINA^-/-^FAIM2^-/-^ mice | MCAO 30min | EPO | 5000 U/kg | Intraperitoneal | 0, 24 and 48h after MCAO | 72h | Upregulated GRINA and FAIM2 mRNA levels, decreased infarct sizes and abrogated neurological impairments in wildtype controls.  GRINA and/or FAIM2 deficient mice showed increased expression levels of cleaved-caspase 3 and of pro-apoptotic BAX mRNA. | EPO-mediated neuroprotection after ischemic stroke involving different caspases and was conveyed by GRINA and FAIM2. | (Habib et al., 2019b) |
| FAIM2^-/-^ and wild type mice | MCAO 30min | EPO | 5000 and 90 000 U/kg | Intraperitoneal | Before (30 min) and after (24 and 48h) MCAO | 72h | In WT mice, EPO at a low dose (5000 U/kg) reduced stroke volume, whereas high dose (90 000 U/kg) did not. In Faim2^-/-^ animals, low-dose EPO did not reduce stroke volume. | Faim2 up-regulation may contribute to the neuroprotective effects of low-dose erythropoietin in transient brain ischemia. | (Komnig et al., 2018) |
| Postnatal day 7 (P7) SOD-tg (CD1) mice | Hypoxic-ischemic brain injury | EPO | 5 U/g | Intraperitoneal | 0, 24h, and 5d after ischemia | 7d | Cannot ameliorate the damage seen in situations where there is excess H2O2 accumulation. | May exacerbate injury in settings of extreme oxidative stress. | (Sheldon et al., 2017) |
| C57BL/6 mice | MCAO 45min | EPO | 5000 IU/kg | Intraperitoneal | Every other day after reperfusion | 14d | Reduced brain tissue loss volume, ameliorated white matter injury, and improved neurobehavioral outcomes. | Attenuated gliosis and facilitated the microglial polarization toward the beneficial M2 phenotype to promote oligodendrogenesis. | (Wang et al., 2017a) |
| Sprague-Dawley rats | MCAO 50min | EPO; EPO+cyclosporine (CsA) | EPO (5,000 IU/kg);  CsA (20 mg/kg) | EPO (subcutaneous);  CsA (intra-peritoneal) | 0.5, 24, and 48h | 72h | Reduced brain infarct area. | Suppressed the innate immune response to inflammation, oxidative stress, microRNAs (miR-223/miR-30a/miR-383) and MAPK family signaling. | (Yuen et al., 2017) |
| Postnatal day 10 (P10) Sprague-Dawley rats | MCAO 3h | EPO | 1000 U/kg per dose×3 doses | intra-peritoneal | Starting one week after MCAO (at P17, P20, and P23) | 30d | Reduced infarction volume, improved both behavioral and histological outcomes. | Not studied. | (Larpthaveesarp et al., 2016) |
| Sprague-Dawley rats | MCAO 2h | EPO, rtPA | EPO (800 U/kg); rtPA (10 mg/kg) | EPO (middle cerebral artery); rtPA (tail vein) | Onset of reperfusion | 24h | Attenuated neurobehavioral deficits, reduced brain infarct and edema volume, TUNEL-positive cells, and downregulated Claudin-5 and Occludin expression. | Not studied. | (Wang et al., 2016b) |
| Sprague Dawley rats | MCAO | EPO | At a dose of 800 U/kg | Middle cerebral artery | At the beginning of reperfusion | 2 and 24h | Alleviated infarct volume, brain edema, and improved neurobehavioral outcomes. | Prevented degradation of Claudin-5 and Occludin, and reduced the expression and activity of MMP-2 and MMP-9 in isolated brain microvessels. | (Wang et al., 2015) |
| Wistar rats | MCAO 1h | EPO | 1000 IU/kg | Intravenous | 10min before the onset of MCAO | 24h | Attenuated severity of neurological deficit and amount of brain edema, did not affect infarct volume. | Related to an indirect effect on brain edema without neuronal anti-apoptotic effects. | (Ratilal et al., 2014) |
